# Characteristics of helicopter emergency medical services (HEMS) dispatch cancellations during a six-year period in a Dutch HEMS region

**DOI:** 10.1186/s12873-021-00439-x

**Published:** 2021-04-16

**Authors:** E. Berkeveld, T. C. N. Sierkstra, P. Schober, L. A. Schwarte, M. Terra, M. A. de Leeuw, F. W. Bloemers, G. F. Giannakopoulos

**Affiliations:** 1 Department of Trauma Surgery, Amsterdam UMC location VUmc, De Boelelaan 1117, 1081 HV Amsterdam, The Netherlands; 2 Department of Anesthesiology, Amsterdam UMC location VUmc, De Boelelaan 1117, 1081 HV Amsterdam, The Netherlands; 3 Helicopter Emergency Medical Service (HEMS) Life Liner One, Amsterdam, The Netherlands; 4 Department of Trauma Surgery, Amsterdam UMC location AMC, Meibergdreef 9, 1105 AZ Amsterdam, The Netherlands

**Keywords:** Helicopter emergency medical services (HEMS), Cancellation, Dispatch, Trauma, Mechanism of injury (MOI)

## Abstract

**Background:**

For decades, Helicopter Emergency Medical Services (HEMS) contribute greatly to prehospital patient care by performing advanced medical interventions on-scene. Unnecessary dispatches, resulting in cancellations, cause these vital resources to be temporarily unavailable and generate additional costs. A previous study showed a cancellation rate of 43.5% in our trauma region. However, little recent data about cancellation rates and reasons exist, despite revision of dispatch protocols. This study examines the current cancellation rate in our trauma region over a six-year period. Additionally, cancellation reasons are evaluated per type of dispatch and initial incident report, upon which HEMS is dispatched.

**Methods:**

This retrospective study analyzed the data of the Dutch HEMS Lifeliner 1 (North-West region of the Netherlands, covering a population of 5 million inhabitants), analyzing all subsequent cases between April 1st 2013 and April 1st 2019. Patient characteristics, type of dispatch (primary; based on dispatcher criteria versus secondary, as judged by the first ambulance team on site), initial incident report received by the EMS dispatch center, and information regarding day- or nighttime dispatches were collected. In case of cancellation, cancel rate and reason per type of dispatch and initial incident report were assessed.

**Results:**

In total, 18,638 dispatches were included. HEMS was canceled in 54.5% (95% CI 53.8–55.3%) of cases. The majority of canceled dispatches (76.1%) were canceled because respiratory, hemodynamic, and neurologic parameters were stable. Dispatches simultaneously activated with EMS (primary dispatch) were canceled in 58.3%, compared to 15.1% when HEMS assistance was requested by EMS based on their findings on-scene (secondary dispatch). A cancellation rate of 54.6% was found in trauma related dispatches (*n* = 12,148), compared to 52.2% in non-trauma related dispatches (*n* = 5378). Higher cancellation rates exceeding 60% were observed in the less common dispatch categories, e.g., anaphylaxis (66.3%), unknown incident report (66.0%), assault with a blunt object (64.1%), obstetrics (62.8%), and submersion (61.9%).

**Conclusion:**

HEMS cancellations are increased, compared to previous research in our region. Yet, the cancellations are acceptable as the effect on HEMS’ unavailbility remains minimized. Focus should be on identifying the patient in need of HEMS care while maintaining overtriage rates low. Continuous evaluation of HEMS triage is important, and dispatch criteria should be adjusted if necessary.

## Background

Helicopter Emergency Medical Services (HEMS) are increasingly used to provide specialized medical care in the out-of-hospital setting [1–8]. For the severely injured patients, HEMS were shown to have an additional survival benefit [1–4]. In the Netherlands, HEMS exist since 1995 and have the main purpose of assisting Emergency Medical Services (EMS) on-scene. Dispatches can coincide with EMS, based on information received by the EMS dispatch center (primary dispatch) often provided by a layperson, or upon request by EMS, based on their findings on-scene (secondary dispatch).

As the availability of specialized lifesaving care is considered imperative, it is pursued to maintain HEMS undertriage as low as possible, with an undertriage rate below 5% considered acceptable according to the American College of Surgeons (ACS) [9]. Efforts to identify the severely injured requiring HEMS assistance were made by previous studies [10, 11], deducing predictors of major trauma based on criteria related to the mechanism of injury (MOI), physiologic parameters, and injury anatomy [10].

HEMS overtriage, resulting in dispatch cancellation, causes vital resources to be temporarily unavailable and generate additional costs. Besides, each dispatch constitutes a risk for the HEMS crew flying by helicopter. Yet, a certain amount of overtriage remains unavoidable [9]. A cancellation rate of 43.5% has been found by a previous study in our HEMS region [12]. Additionally, cancellations were more frequent in incidents where the mechanism of trauma was minor, and the injury was located at the extremities, compared to dispatches that resulted in an arrival at the scene [10, 12]. However, no recent empirical data exists despite HEMS increasing experience and renewal of dispatch protocols. New insights in our cancellation rate and reasons for cancellation might contribute to the optimization of HEMS triage.

This study aimed to examine the current cancellation rate in our trauma region over a six-year period. Reasons for cancellation were evaluated per type of dispatch (primary versus secondary dispatch) and per initial incident report upon which HEMS is dispatched. We hypothesized that the cancellation rate would be lower compared to previous studies because of iterative improvement of dispatch questionnaire script over the last 10 years and increased experience with HEMS involvement in the prehospital setting.

## Methods

### Study setting

In the Netherlands, prehospital advanced medical and interventional trauma care is provided on a 24-h, 7 days a week basis by EMS and four additional HEMS services. The main purpose of HEMS is to provide a specialized, physician-based team on-scene that can perform additional lifesaving care such as advanced airway management, administration of specialized medication, blood products, and provide selected surgical interventions (including resuscitative thoracotomy, chest tube placement, surgical airway and amputation of extremities). Given the short distance to the trauma centers on average, patient transportation by helicopter only occurs occasionally, as the HEMS physician accompanies the patient in the ambulance during transport to the trauma center [13].

The Dutch HEMS crews consist of a HEMS physician (trauma surgeon or anesthesiologist), HEMS nurse (Emergency Department’s (ED) nurse, or EMS nurse who acquired special training in navigating and assisting the pilot as a HEMS Crew Member (HCM)), HEMS pilot and chauffeur. Depending on weather conditions or scene access, a chauffeur is used to transport the crew in the rapid response vehicle.

### Dispatch and cancellation

HEMS dispatch can occur either as a primary dispatch, in which HEMS is dispatched simultaneously with EMS, based on a layperson’s call to the EMS dispatch center, or as a secondary dispatch when assistance is requested by the EMS crew already on-scene (e.g., the situation is worse than initially appeared or assistance with tracheal intubation is required).

HEMS triage is performed by the EMS dispatch center’s centralist, a specially trained nurse, who, after receiving the initial call, can activate a HEMS dispatch request according to a systematic triage protocol. Primary dispatches are often based on a description of the mechanism of injury (MOI) or pronounced pathophysiologic or anatomical abnormalities. Also, an additional lower threshold is adhered to when incidents concern a child’s involvement [14].

Once EMS has arrived on-scene, they provide a situation report through a continuous line to the EMS dispatch center’s centralist and the already dispatched HEMS crew. Based on EMS’s clinical judgment and experience, they could state that HEMS assistance is no longer required. Subsequently, the HEMS physician ultimately decides whether to cancel a dispatch, taking into account the HEMS cancellation criteria [10]. In general, a dispatch is canceled in case of respiratory, hemodynamic, and neurologic stable parameters with no expected physiologic deterioration within one hour, an indication for “Scoop and Run” to the nearest trauma center, a patient already being deceased, or a false incident report [14].

For the patient to receive hospital-level care as soon as possible, HEMS’ duration to arrive at the scene versus EMS’ duration to transport the patient to a hospital is under constant consideration. An option to limit the time spent on-scene is by arranging a rendezvous between EMS and HEMS, in which the HEMS physician joins in the patient and EMS during transportation in the ambulance. Sometimes, arrival at the hospital would be faster than HEMS would take to arrive at the patient, then a joint decision between EMS and HEMS is made to cancel the HEMS dispatch. The HEMS physician could still provide treatment advice if contributing.

### Study design and data extraction

This retrospective study analyzed all data of the Dutch HEMS Lifeliner 1 (Trauma Region North West Netherlands covering a population of about five million inhabitants). Patient characteristics, type of dispatch, and initial incident report received by the EMS dispatch center were collected. Additionally, in case of a canceled dispatch, the reason for- and time of cancellation was obtained. Time until cancellation was calculated as the difference between the time of cancellation and HEMS dispatch time. Times exceeding 30 min without logical explanation were excluded from the analysis due to the suspicion of outliers by data entry errors. Dispatches without resulting information regarding arrival at the scene or cancellation were also excluded from the analysis. Cancellation rates and reasons were calculated for each type of dispatch and initial incident report.

### Statistical analysis

Descriptive statistics were used. Cancellation rates were presented as percentages and 95% confidence intervals (CI), whereas continuous variables were presented as median with interquartile range (25th to 75th percentile). Data were analyzed using IBM SPSS Statistics version 24.0 (IBM, New York, USA).

## Results

**Fig. 1:**
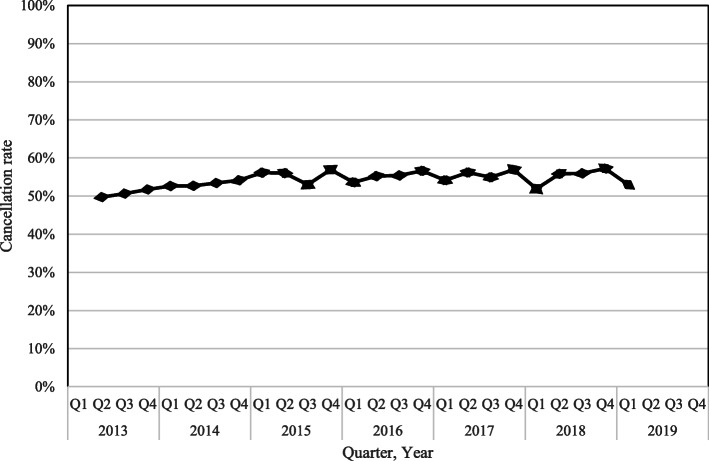
HEMS cancellation rate over the years

In total, out of 18,706 HEMS dispatches, 18,638 were eligible for inclusion. Dispatches with missing data concerning whether the dispatch resulted in an arrival at the scene or a cancellation (*n* = 68) were excluded from the analysis. Overall, a cancellation rate of 54.5% (*n* = 10,166; 95% CI 53.8–55.3%) was found, compared to 45.5% (*n* = 8472; 95% CI 44.7–46.2%) for dispatches resulting in arrival at the scene. Over the examined years, a relatively stable cancellation rate was found (Fig. 1).

### Type of dispatch

Of all dispatches, the vast majority consisted of primary dispatches (*n* = 16,704; 89.6%) compared to secondary dispatches (*n* = 1695; 9.1%) and in 1.3% (*n* = 239) data was missing regarding type of dispatch. A mean cancellation rate of 58.3% (*n* = 9731; 95% CI 0.575–0.590) was found for primary dispatches compared to 15.1% (*n* = 256; 95% CI 0.134–0.169) for secondary dispatches.

**Fig. 2:**
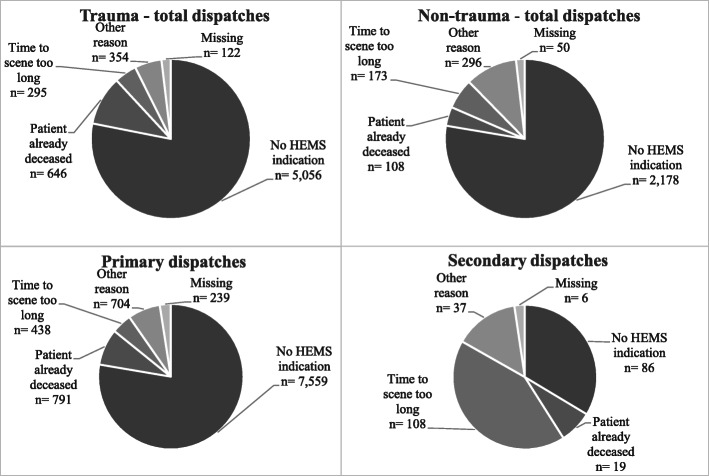
Reason for dispatch cancellation per type of dispatch

Figure 2 illustrates the different reasons for canceled dispatches. For the total study population, “No HEMS indication,” which refers to stable respiratory, hemodynamic, and neurologic parameters in a patient, was the most common cancellation reason (*n* = 7733; 76.1%). Stratified by dispatch-type, “no HEMS indication” was the main reason for cancellation for primary dispatches (*n* = 7559; 77.7%), whereas “anticipated HEMS transport time to scene too long” was most common in secondary dispatches (*n* = 108; 42.2%).

### Initial incident reports

As shown in Table 1, the majority of initial incident reports were trauma related (*n* = 12,148; 65.2%) compared to non-trauma related (*n* = 5378; 28.8%) and unknown (*n* = 1112; 6.0%), the latter in which no description was mentioned in the report. Specifically, dispatches concerning incidents involving “fall from height” (*n* = 3485; 18.7%), “respiratory, hemodynamic or neurologic instability” (*n* = 2007; 10.8%) or “unspecified traumatic incident” (*n* = 1715; 9.2%) were most common. Highest cancellation rates were seen in “anaphylaxis” (*n* = 285; 66.3%), “unknown incident report” (*n* = 734; 66.0%) or “assault with a blunt object” (*n* = 123; 64.1%). Lowest cancellation rates were found in incidents involving “unspecified non-traumatic incident” (*n* = 21; 29.2%), “intoxication” (*n* = 58, 30.4%) and “assault with a firearm” (*n* = 112; 40.3%).

**Table 1:** Frequencies and cancellation rate per initial incident report

Initial incident report	Frequency of all dispatches, No. (%)	Cancellation per initial incident report. No. (%)
**Trauma**	12,148 (65.2%)	6627 (54.6%)
Pedestrian accident	704 (3.8%)	395 (56.1%)
Bicycle accident	1487 (8.0%)	795 (53.5%)
Scooter accident	598 (3.2%)	287 (48.0%)
Motorcycle accident	365 (2.0%)	158 (43.3%)
Motor vehicle accident	1175 (6.3%)	597 (50.8%)
Fall from height	3485 (18.7%)	2013 (57.8%)
Assault	1279 (6.9%)	621 (48.6%)
- Blunt	192	123 (64.1%)
- Stabbing	809	386 (47.7%)
- Firearm	278	112 (40.3%)
Heavy object on body	148 (0.8%)	86 (58.1%)
Entrapment	295 (1.6%)	145 (49.2%)
Strangulation	464 (2.5%)	272 (58.6%)
Blast, fire or chemical Injury	433 (2.3%)	231 (53.3%)
Unspecified traumatic Incident	1715 (9.2%)	1027 (59.9%)
**Non-trauma**	5378 (28.8%)	2805 (52.2%)
Advanced airway management required	748 (4.0%)	335 (44.8%)
Respiratory, hemodynamic or neurologic instability	2007 (10.8%)	927 (46.2%)
Anaphylaxis	430 (2.3%)	285 (66.3%)
Intoxication	191 (1.0%)	58 (30.4%)
Submersion	1525 (8.2%)	944 (61.9%)
Obstetrics	86 (0.5%)	54 (62.8%)
Neonatal resuscitation	319 (1.7%)	181 (56.7%)
Unspecified non-traumatic incident	72 (0.4%)	21 (29.2%)
**Unknown**	1112 (6.0%)	734 (66.0%)
Unkown report	1112 (6.0%)	734 (66.0%)

The major reason for dispatch cancellation in all initial incident reports was “No HEMS indication” (Table **2**). In contrast, for incidents involving “pedestrian accident” or “strangulation,” the main reason for cancellation was a “patient already deceased,” 42.8% (*n* = 169) and 64.0% (*n* = 174), respectively. In the report category “pedestrian accident,” 40% (*n* = 150) of cancellations concerned pedestrian accidents with involvement of a train.

**Table 2:** Reasons for cancellation per initial incident report

Initial incident report	Cancellation reason					
	No HEMS indication No. (%)	Patient already deceased No. (%)	Time to scene too long No. (%)	HEMS dispatch impossible No. (%)	Other reason No (%)	Missing No (%)
**Trauma**						
Pedestrian accident	173 (43.8%)	169 (42.8%)	18 (4.6%)	2 (0.5%)	24 (6.1%)	9 (2.3%)
Bicycle accident	691 (86.9%)	12 (1.5%)	50 (6.3%)	8 (1.0%)	24 (3.0%)	10 (1.3%)
Scooter accident	248 (86.4%)	9 (3.1%)	12 (4.2%)	2 (0.7%)	12 (4.2%)	4 (1.4%)
Motorcycle accident	124 (78.5%)	13 (8.2%)	9 (5.7%)	0	9 (5.7%)	3 (1.9%)
Motor vehicle accident	475 (79.6%)	55 (9.2%)	18 (3.0%)	4 (0.7%)	37 (6.2%)	8 (1.3%)
Fall from height	1743 (86.6%)	114 (5.7%)	63 (3.1%)	9 (0.4%)	50 (2.5%)	34 (1.7%)
Assault	439 (70.7%)	33 (5.3%)	66 (10.6%)	1 (0.2%)	69 (11.1%)	13 (2.1%)
- Blunt	116 (94.3%)	0	3 (2.4%)	1 (0.8%)	3 (2.4%)	0
- Stabbing	276 (71.5%)	7 (1.8%)	51 (13.2%)	0	43 (11.1%)	9 (2.3%)
- Firearm	47 (42.0%)	26 (23.2%)	12 (10.7%)	0	23 (20.5%)	4 (3.6%)
Heavy object on body	75 (87.2%)	0	2 (2.3%)	0	7 (8.1%)	2 (2.3%)
Entrapment	111 (76.6%)	18 (12.4%)	8 (5.5%)	0	4 (2.8%)	4 (2.8%)
Strangulation	64 (23.5%)	174 (64.0%)	6 (2.2%)	2 (0.7%)	21 (7.7%)	5 (1.8%)
Blast, fire or chemical Injury	154 (66.7%)	9 (3.9%)	29 (12.6%)	4 (1.7%)	31 (13.4%)	4 (1.7%)
Unspecified traumatic incident	831 (80.9%)	53 (5.2%)	62 (6.0%)	6 (0.6%)	45 (4.4%)	30 (2.9%)
**Non-trauma**						
Advanced airway management required	271 (80.9%)	13 (3.9%)	25 (7.5%)	2 (0.6%)	20 (6.0%)	4 (1.2%)
Respiratory, hemodynamic or neurologic instability	719 (77.6%)	38 (4.1%)	82 (8.8%)	12 (1.3%)	59 (6.4%)	17 (1.8%)
Anaphylaxis	224 (85.6%)	0	21 (7.4%)	2 (0.7%)	14 (4.9%)	4 (1.4%)
Intoxication	45 (77.6%)	3 (5.2%)	5 (8.6%)	1 (1.7%)	4 (6.9%)	0
Submersion	703 (74.5%)	47 (5.0%)	9 (1.0%)	7 (0.7%)	160 (16.9%)	18 (1.9%)
Obstetrics	27 (50.0%)	2 (3.7%)	17 (31.5%)	0	4 (7.4%)	4 (7.4%)
Neonatal resuscitation	157 (86.7%)	5 (2.8%)	12 (6.6%)	0	4 (2.2%)	3 (1.7%)
Unspecified non-traumatic incident	12 (57.1%)	0	2 (9.5%)	0	7 (33.3%)	0
**Unknown**						
Unknown report	427 (58.2%)	50 (6.8%)	42 (5.7%)	26 (3.5%)	83 (11.3%)	106 (14.4%)

Overall, time from HEMS alarm to cancel, including both dispatches facilitated by helicopter and rapid response vehicle, was available in 95.9% (*n* = 9745) of dispatches. Median time until cancellation was 7 min (IQR 5–10). Specifically, median airborne time, indicated as the time from helicopter departure until cancellation, was available in 89.3% (*n* = 9083) of helicopter facilitated dispatches. Median airborne time was 5 min (IQR 3–7).

In total, 64.9% (*n* = 12,095) dispatches were performed during daylight, compared to 35.1% (*n* = 6543) dispatches being performed at nighttime. The cancellation rate for daylight dispatches was 54.5% (*n* = 6591), compared to 54.6% (*n* = 3575) for nighttime dispatches.

Dispatches for incidents involving newborns and babies, aged between zero until 1 year of age (1.1%, *n* = 208) showed a cancellation rate of 60.6% (*n* = 126). Children under the age of 18 were involved in 16.4% (*n* = 3065) of dispatches and had a cancellation rate of 61.0% (*n* = 1870). Dispatches involving adult patients (53.6%, *n* = 9992) were most common and showed a cancellation rate of 46.6% (*n* = 4659.). In 240 cases (1.3%) patient age data was missing.

## Discussion

Availability of HEMS for patients in need of their care is essential. According to the ACS, an overtriage level up to 35% is accepted to keep undertriage below 5% concerning the in-hospital setting [9]. However, for HEMS systems, the necessity to accept a certain amount of cancellations due to overtriage in order to ensure low levels of undertriage might apply as well. In the literature, various rates of overtriage were found for different patient categories [15–17]. This study aimed to provide insight into a Dutch HEMS region’s cancellation characteristics by analyzing a large cohort of more than 18,000 dispatches. In the total study population, a mean cancellation rate of 54.5% was found.

The cancellation rate found in this study is increased compared to previous research in this HEMS region, as Giannakopoulos et al. have found a cancellation rate of 43.5% in data originating from 2006 [12]. Despite the current study’s hypothesis that increasing experience would cause a lower cancellation rate, the opposite finding might be explainable as well. HEMS’ fast and dynamic development could have caused a noticeable cancellation increase over the years. Their added value was scientifically demonstrated, and their presence at the scene is more established [1, 3, 4, 18–22]. Therefore, a lower threshold is adhered to utilizing HEMS, consequently increasing the cancellation rate. In our study’s dataset, a relatively stable cancellation rate was found over the examined years. These rather stable values contrast the findings of a previous study conducted in a different Dutch HEMS region by Gerritse et al. They found a steady increase in cancellation rate from 36 to 54% between 2001 and 2008 [23]. Therefore, the current stable cancellation rate might be caused by the increased maturation of HEMS in the prehospital system, consequently maintaining cancellation rates over time relatively stable.

### Primary versus secondary dispatches

Primary dispatches were most common and showed a relatively high cancellation rate of 58.3%, compared to secondary dispatches in which a cancellation rate of 15.1% was found. Similar results have been found by McQueen et al. concerning medical-related dispatches, showing a higher cancellation rate for primary dispatches (26.2%) than secondary dispatches (8.4%) in a UK HEMS system [24]. The difference between primary and secondary dispatches was anticipated, as the decision for primary dispatch is often based on information provided by a layperson [9]. Therefore, the EMS dispatch center adheres to a low dispatch threshold for primary dispatches. Cancellation of a dispatch occurs by EMS as soon as they consider HEMS assistance unnecessary (or in case a scoop-and-run is more feasible than waiting for HEMS). This is in contrast to secondary dispatches, wherein HEMS assistance is requested by EMS themselves because additional assistance on-scene is required - and this makes it less likely that they will subsequently cancel it. However, in some cases, HEMS’ assistance is initially requested by EMS, while soon after, the patient’s respiratory, hemodynamic and neurologic parameters stabilize (e.g., as a response to treatment provided by EMS). This could explain the current study’s cancellations for the reason of ‘no HEMS indication’ in secondary dispatches.

### Triage

Previous studies contributed to HEMS system’s improvement by examining measures to optimize HEMS triage. An earlier study conducted in our HEMS region identified predictors for major trauma and, with that, contributed to improvement of the triage algorithm [10]. Major trauma patients often show both anatomical injury and abnormalities in vital signs. For this reason, a combined MOI description, physiologic parameters, and anatomical injury would provide the most accurate prediction of HEMS requirement [12, 14]. Moreover, besides algorithms for a sensitive and specific dispatch protocol [25], we assume that an essential aspect in reducing overtriage concerns familiarity with criteria and, above all, the professionals’ experience and clinical judgment. Studies showed that increased practice and familiarity with dispatch criteria could reduce overtriage [19, 26–28]. Therefore, training of dispatch centralists, EMS – and HEMS crews might contribute to minimization of overtriage.

To the best of our knowledge, no previous study assessed the cancellation characteristics for various types of incidents in such detail. Incidents involving anaphylaxis, unknown incident report, or assault with a blunt object showed the highest cancellation rates. The lowest rates were seen in incidents involving an unspecified non-trauma incident, intoxication, and assault with a firearm. The majority of dispatches were canceled because a patient’s physiologic, hemodynamic, and neurologic parameters were stable, which is in line with previous research [12, 23]. Noticeably, considering penetrating injury, most cancellations were for the reason ‘time to scene too long’. Therefore, it might be indicating a positive sign of patient-orientated decision-making.

### Incidents with pediatric involvement

In this study, 17.5% of dispatches involved a child (below 18 years). Compared to incidents involving adult patients, this group had a higher cancellation rate, 61.0% compared to 46.6%, respectively. In contrast, a lower cancellation rate for pediatric involvement of 27% was found in another Dutch HEMS region [23]. Concerning incidents involving a child, it was shown that HEMS have an increased success rate for Advanced Life Support restricted procedures, and an additional 2.5 lives are saved per 100 dispatches [18, 20, 29]. However, identifying children in need of acute trauma care remains challenging, as van der Sluijs et al. showed an undertriage rate of 16.3% based on data derived from several Dutch trauma regions [30]. Moors et al. showed in a different Dutch HEMS system that the variables Glasgow Coma Scale (GCS), Injury Severity Score, systolic blood pressure, and respiratory rate might serve as good predictors for mortality in pediatric trauma patients in the out-of-hospital setting [18]. However, none of them is available at a primary HEMS dispatch. Therefore, adhering to a low dispatch threshold for incidents involving a child is recommended [6, 14, 31]. Even more, a patient’s neurologic state was already identified as a triage criterion, with a sensitivity of 97.9% and a specificity of 96% for using GCS [15, 23]. Besides, a neurologic triage criterion might be estimated roughly by a layperson, making it more applicable in the prehospital setting.

### Cancellation costs

HEMS’ cost efficiency has already been demonstrated in (inter-)national literature [12, 32, 33]. In the Netherlands, a HEMS crew is available on a 24-h, 7 days a week basis. Therefore, variable costs are influenced by a canceled dispatch, whereas HEMS’ sunk costs remain unaffected. In this study, despite a cancellation rate of 54.5%, the median (one-way) airborne time of 5 min and an average of 5 cancellations per 24-h contribute to 50 extra flight minutes per 24-h due to cancellations. Besides, on average, only 2/3 of dispatches are facilitated by helicopter, compared to 1/3 of dispatches in which the rapid response vehicle is used. However, as there is a window of inoperability to other patients requiring HEMS care concomitantly, opportunity costs are also involved in a dispatch cancellation. To overcome this, the dispatch center is notified when a cancellation is made during the flight, making HEMS immediately available for another incoming dispatch request. Therefore, the unavailability of HEMS is minimized. Moreover, the intervention of HEMS teams constitutes a risk due to the use of a helicopter. Optimization of overtriage would therefore also create possibilities to limit this risk.

In our HEMS system, even though the cancellation rate is increased compared to previous studies, the level of overtriage seems acceptable. We believe that the priority is to maintain low undertriage and ensure that overtriage stays within reasonable limits. Focus should be in particular on the effect that overtriage may have on HEMS’ availability. As our system is characterized by a fast time until cancel, with limited canceled dispatches per 24-h and confined related cancellations costs, we believe that the current cancellation rate is tolerable. That being said, continuous critical evaluation of the triage criteria and the consequences of overtriage remains vital to secure optimal efficiency of the HEMS system.

### Limitations

This study’s strengths are the large number of included dispatches and the duration of the study period, creating a substantial amount of data available. However, there are also some limitations. The retrospective descriptive design provides a lower level of evidence than a well organized randomized controlled trial or prospective design. However, for this study question, a randomized design would not be possible. Second, no information was available regarding “false negative” dispatches wherein dispatches were canceled, but HEMS could still have contributed. Unfortunately, variables that indicate severe instability are scarce and often not well recorded in prehospital databases. Future research could focus on examining the over-and undertriage per initial incident report. This, in order to consider per initial incident report whether HEMS dispatch would be accurate and possibly contribute to a revision of triage criteria.

## Conclusion

HEMS cancellation rates have been stable for the last 6 years, however, this current plateau is considerably higher than the cancellation rates 10–15 years ago. Constant focus should be the identification of patients in need of HEMS care while maintaining overtriage rates low. Consequences of overtriage, such as HEMS’ unavailability and additional costs, should be frequently evaluated. Dispatches for incidents involving pediatric patients had rather high cancellation rates, while trauma and non-trauma dispatch cancellation rates were similar. Continuous evaluation of HEMS triage is important, and dispatch criteria should be regularly adjusted in a data-driven manner.

## Data Availability

Original data remain available and access may be provided upon reasonable request.
